# Human sperm proteome reveals the effect of environmental borne seminal polyaromatic hydrocarbons exposome in etiology of idiopathic male factor infertility

**DOI:** 10.3389/fcell.2023.1117155

**Published:** 2023-05-16

**Authors:** Jasmine Nayak, Soumya Ranjan Jena, Sugandh Kumar, Sujata Kar, Anshuman Dixit, Luna Samanta

**Affiliations:** ^1^ Redox Biology & Proteomics Laboratory, Department of Zoology, Ravenshaw University, Cuttack, India; ^2^ Center of Excellence in Environment & Public Health, Ravenshaw University, Cuttack, India; ^3^ Institute of Life Sciences, NALCO Square, Bhubaneswar, India; ^4^ Kar Clinic and Hospital Pvt., Ltd., Unit-IV, Bhubaneswar, India

**Keywords:** idiopathic male infertility, polyaromatic hydrocarbon (PAHs), proteomics, oxidative stress, AhR signaling, protein nitrosylation

## Abstract

**Introduction:** Polyaromatic hydrocarbons (PAHs) are considered as redox active environmental toxicants inducing oxidative stress (OS) mediated injury to cells. Oxidative predominance is reported in 30%–80% of idiopathic male infertility (IMI) patients. Hence, this work aims to unravel correlation, if any, between seminal PAH exposome and sperm function in IMI patients through a proteomic approach.

**Methods:** Seminal PAH exposome was analyzed in 43 fertile donors and 60 IMI patients by HPLC and receiver operating characteristic (ROC) curve was applied to find out the cut-off limits. Spermatozoa proteome was analyzed by label free liquid chromatography mass spectroscopy (LC-MS/MS) followed by molecular pathway analysis using bioinformatic tools. Validation of key proteins’ expression and protein oxidative modifications were analyzed by western blot.

**Results and discussion:** Of the 16 standards toxic PAH, 13 were detected in semen. Impact of the different PAHs on fertility are Anthracene < benzo (a) pyrene < benzo [b] fluoranthene < Fluoranthene < benzo (a) anthracene <indol (123CD) pyrene < pyrene < naphthalene < dibenzo (AH) anthracene < fluorene < 2bromonaphthalene < chrysene < benzo (GH1) perylene as revealed by ROC Curve analysis (AUC_ROC_). Benzo [a] pyrene is invariably present in all infertile patients while naphthalene is present in both groups. Of the total 773 detected proteins (Control: 631 and PAH: 717); 71 were differentially expressed (13 underexpressed, 58 overexpressed) in IMI patients. Enrichment analysis revealed them to be involved in mitochondrial dysfunction and oxidative phosphorylation, DNA damage, Aryl hydrocarbon receptor (AHR) signaling, xenobiotic metabolism and induction of NRF-2 mediated OS response. Increased 4-hydroxynonenal and nitrosylated protein adduct formation, and declined antioxidant defense validates induction of OS. Increased GSH/GSSG ratio in patients may be an adaptive response for PAH metabolism via conjugation as evidenced by over-expression of AHR and Heat shock protein 90 beta (HSP90β) in patients. Seminal PAH concentrations, particularly benzo (a) pyrene can be used as a marker to distinguish IMI from fertile ones with 66.67% sensitivity and 100% specificity (95% confidence interval) along with oxidative protein modification and expression of AHR and HSP90β.

## 1 Introduction

Semen quality of men in their reproductive age is markedly deteriorating over the past decades (Carlsen et al., 1992; Guzick et al., 2001). Approximately 15% of the co-inhabiting couples are infertile where 50% of them have abnormal semen parameters implying the involvement of male-infertility-associated factor (Jungwirth et al., 2012). Male infertility in general is known as a multi-causal effect with a very few large-scale epidemiological reports available (Pierik et al., 2000; Tuttelmann et al., 2011). Due to the paucity of information on causative factors on decline in semen quality; accurate diagnosis and personalized treatment options are restricted. An infertility case with unknown causative factor is identified and referred to as idiopathic infertility (de Kretser, 1997; Jungwirth et al., 2012). It is reported that ∼75% of oligospermic men are idiopathic (Punab et al., 2017). Albeit, the interplay between genetic, environmental and lifestyle factors are proposed to be behind this condition; very few reports established the role of environmental toxins in idiopathic infertility (Krzastek et al., 2020). Most of the studies simply establish a correlation between environmental toxin levels in body fluids such as blood plasma or urine with semen parameter without giving much insight into the mechanisms involved (Krzastek et al., 2020; Zhu et al., 2021). European Association of Urology attributed idiopathic male factor infertility to endocrine disruption due to environmental pollution, reactive oxygen species, or genetic abnormalities (Sharman, 2017). In recent times, more emphasis is given to the “exposomes” concept that refers to the totality of environmental exposures of an individual during the lifetime. This novel approach combines the body burden of environmental toxins and modern omics technologies to study the role of the environment in human diseases (Vineis et al., 2020). Many environmental toxins such as pesticides, herbicides, phthalates and polyaromatic hydrocarbons (PAHs) undergo metabolic activation in human body and cause oxidative stress (Baulig et al., 2003). Oxidative stress is time and again reported to not just correlate with defective sperm function but is causally involved in the genesis male factor infertility (Agarwal et al., 2006; Aitken, 2016; Wagner et al., 2018). Male Oxidative Stress Infertility (MOSI) is proposed for the management of idiopathic male infertility with measurement of seminal oxidation-reduction potential (ORP) as an easy clinical biomarker (Agarwal et al., 2019). It is further suggested that upto 80% of the total cases of idiopathic infertility have augmented oxidative stress (Agarwal et al., 2006; Aitken, 2016; Wagner et al., 2018). Therefore, it is imperative to look into the cause behind the etiology of MOSI in idiopathic infertility as a function of environmental toxins.

The crucial environmental toxins, especially phthalates, bisphenols, pesticides, flame retardants and PAHs warrant special attention due to their potential role as endocrine disruptors affecting hypothalamo-pituitary-thyroid axis and hypothalamo-pituitary-gonadal axis (Maric et al., 2021). PAHs are the by-products of incomplete combustion of organic materials generated from tobacco and cigarette smoke, barbequed food, vehicle exhaust and oil spillers as well as during coke production and chemical manufacturing. They are usually metabolically activated by cytochrome P450 enzymes during steroidogenesis and promotes free radical generation (Hanukoglu, 2006). The ROS (H_2_O_2_ and O_2_^·−^) generated during normal steroidogenesis are within critical levels and play an important role in the regulation of steroidogenic activity of the Leydig cell (Tai and Ascoli, 2011). The elevated production of ROS have been found to inhibit steroid productions, and causes damage to mitochondrial membrane of spermatozoa (Luo et al., 2006). However, our knowledge on oxidative stress-induced idiopathic male infertility as a function of environmental borne seminal concentration of PAH is extremely limited in general and with respect to non-occupational exposure in particular. With this background, the present study in designed to find out the level of PAH and oxidative stress in the ejaculate of idiopathic infertile men and the mechanism of sperm dysfunction in these patients via high throughput comparative shotgun proteomic analysis towards discovery of plausible biomarkers.

## 2 Materials and methods

### 2.1 Ethics statement and patient selection

After approval from the Institutional Ethics Committee, patients attending the infertility centre and confirmed fertile donors at Kar Clinic and Hospital Pvt. Ltd., Bhubaneswar, Odisha, India, were recruited for the study. Informed written consent was obtained from all individuals before their participation in the study. The exclusion criteria were leukocytospermia (Endtz positive), azoospermia, history of systemic illness, inflammation of reproductive tract (orchitis, epididymitis, urethritis, and testicular atrophy), sexually transmitted disease, varicocele and medications. The patient group included in the present investigation had at least one abnormal semen parameter (i.e., sperm concentration < 15 × 10^6^ sperm/mL, motility < 40%, sperm vitality < 58%, normal sperm morphology < 4%), according to World Health Organization (WHO) 2010 guidelines and were below 40 years of age. The participants included in this study were non-smokers, non-alcoholic, and had a normal body mass index. To ascertain the presence of PAH in semen, 4 smokers from the infertile category were included in the study as positive PAH group since 14 different metabolites of PAH are reported in mainstream cigarette smoke (Vu et al., 2015). Healthy donors (with no known medical condition) who had established fertility recently i.e., within 1 year with no history of pregnancy loss were included as the control group. Subsequently, upon estimation of PAH concentrations, receiver operating characteristic (ROC) curve analysis (as described below) was carried out to establish the cut off level of seminal PAH that differentiates idiopathic infertile patients from fertile donors. A markedly augmented level of all PAH was detected in all four infertile smoker group establishing the presence of PAH metabolites to known exposure. A total of 43 proven fertile donor and 60 idiopathic infertile patients were screened for presence of PAH in the semen and the obtained values were subjected to ROC curve analysis. Of the 43 idiopathic infertile patients, 18 were excluded as they contain lower than cut off value of one or more PAH present in their semen so as to exclude the possibility of factors other than PAH exposure on infertility. Therefore, in the final step 43 proven fertile donor and 42 PAH positive idiopathic infertile patients were included as control and experimental group for comparison of seminogram, measurement of glutathione redox couple and total antioxidant capacity. The Minimum Information about Proteomics Experiment (MIAPE) guidelines of the Human Proteome Organization’s Proteomics Standards Initiative (HUPO-PSI) for reporting proteomics studies was followed for proteome profiling of spermatozoa. Biological variability was maintained in shotgun proteome profiling by including two individual samples and one pooled sample (from 10 randomly picked individuals) and each sample were run in triplicate to maintain technical variability. According to the statistical justification given by Clough et al. (2012), the sample size is justified as per the practice in studies involving high throughput label free LC-MS/MS experiments (Clough et al., 2012; Jena et al., 2021; Moscatelli et al., 2019). The pooling of samples was done on the basis of protein concentration as well as number of spermatozoa per individual so as to have equal contribution from each individual.

### 2.2 Semen analysis

After 3–5 days of sexual abstinence, semen samples were collected from all participants in both groups (idiopathic infertile patients, *n* = 60; fertile donor, *n* = 43) by masturbation. Samples were incubated at 37°C for 20–30 min to achieve complete liquefaction followed by semen analysis according to World Health Organization (WHO) 2010 guidelines (WHO, 2010). Basic semen analysis includes macroscopic (volume, pH, colour, viscosity, liquefaction time) and microscopic (sperm concentration, motility, morphology) along with peroxidase or Endtz test. Samples with > 1.0 × 10^6^/mL round cell with a positive peroxidase test were excluded from the study. After liquefaction and semen analysis, the samples were subjected to centrifugation at 400 *g* for 20 min at 37°C to separate the sperm and seminal plasma. The seminal plasma was processed for PAH measurement and spermatozoa fraction was used for proteomic analysis.

### 2.3 Measurement of seminal PAH exposomes

The HPLC analysis was carried out by injecting 20 μL of the seminal plasma into the chromatographic system (Thermo Scientific UltiMate 3,000) for the determination of PAHs concentration using PAH standards. PAHs were segregated by C-18 column with a gradient elution process using solvent water and acetonitrile. The elution conditions applied were: 0–20 min, 40% of acetonitrile isocratic; 20–37 min, 50%–100% of acetonitrile gradient, 37–42 min, 100% of acetonitrile isocratic, 42–45 min, 100%–40% of acetonitrile, gradient. The flow rate was set at 1 mL/min, at room temperature. Under these conditions, PAHs could be separated satisfactorily within 45 min. The PAHs were identified by comparing the retention time with those of standards taken. The concentration of PAH in semen was calculated according to the formula:

PAH concentration=Peak area of sample/Peak area of standard

### 2.4 Determination of PAH threshold for incidence of infertility

Receiver operating characteristics (ROC) curve is used in clinical biochemistry for the determination of the cut-off point in clinical deterioration. A ROC curve shows a graphical plot illustrating the diagnostic ability of a binary classifiers to identify the sensitivity and the specificity of PAH levels in the prediction of male fertility status. The ROC curves were created using total individual PAHs as “test variables,” and “fertile donor” *versus* idiopathic infertile patients’ (binary variable, with fertile ¼ 0 and infertile patient ¼ 1) as “state variable” and setting the value of the “state variable” as 1. The optimal cut-off value was determined with the use of the Youden index to maximize the sum of sensitivity and specificity.

### 2.5 Sperm protein extraction and estimation

Post separation from seminal plasma, sperm pellet extracted was washed thrice with phosphate buffer saline (PBS) and centrifuged at 400 *g* for 10 min, at 4°C. Sperm lysate was prepared by adding 100 μL of Radio-immunoprecipitation assay (RIPA) buffer supplemented with Protease inhibitor cocktail (cOmplete ULTRA Tablets; Roche) to the sperm pellet and left overnight at 4°C for complete cell lysis. The lysate was centrifuged at 14,000 g for 30 min at 4°C followed by separation of the supernatant. Protein quantification of the supernatant was determined using bicinchoninic acid (BCA; Thermo Fisher Scientific, Waltham, MA).

### 2.6 Assessment of glutathione and redox potential

The total GSH equivalents (GSH + GSSG) were measured spectrophotometrically by 5–5′-dithiobis 2-nitrobenzoic acid (DTNB; Ellman’s reagent) using glutathione reductase (GR) recycling assay (reduction of GSSG to GSH) at the expense of NADPH oxidation. In a similar manner, GSSG was quantified by masking GSH with 2-vinylpyridine. Using Nernst equation, the reduction potential (Ehc) of GSH/GSSG was calculated to determine the cellular oxidative stress status. In brief, the sperm lysate (described above) was deproteinized and precipitated by addition of ice-cold 5% trichloroacetic acid containing 0.01N HCl, and cleared by centrifugation. Glutathione (GSH equivalent and GSSG) estimation was carried out using the deproteinized supernatants. The assay mixture (final volume 200 μL) contained 3 mM NADPH in 125 mM Phosphate buffer containing 6.3 mM EDTA (pH 7.5), DTNB (0.6 mM) and sperm lysate (25 μg protein). To this 2 μL GR (∼1 Unit, Sigma- Aldrich, St. Louis, MO, United States) was added and the yellow chromatophore (2-nitro-5-thiobenzoate: TNB2-) formed by the interaction of -SH groups from GSH and GSSG (after conversion by GR) with DTNB was recorded at 405 nm in a iMark Absorbance Microplate Reader (BioRad Instruments, Inc., Japan) at 1 min intervals for 6 min. All the determinations were normalized to protein content. The absolute GSH amount was quantified from difference between the total GSH equivalent and the obtained GSSG value.

The glutathione redox potential (E^hc^) was calculated by Nernst equation for half reaction: E^hc^ = −240–61.5/2 ln{[GSH]2/GSSG} mV; where −240 mV is the standard redox potential (E^o^) of GSH at pH 0, -61.5/2 denotes RT/z F i.e., R = Gas constant (8.314 J K-1 mol-1), T = absolute temperature of 37°C or 310 K, F= Faraday constant (9.64853 × 104 C mol-1), z = number of electrons exchanged in the chemical reaction GSSG + 2e- + 2H → 2GSH.

### 2.7 Shotgun proteome profiling of spermatozoa

Proteomic analysis was carried out on the isolated spermatozoa proteins using LC-MS/MS. 50 μL of sperm lysate from each sample (*n* = 3 per group; one pooled sample and two individual samples) were taken and reduced with 5 mM TCEP, then alkylated with 50 mM iodoacetamide and subsequently digested with Trypsin (1:50, Trypsin/lysate ratio) for 16 h at 37°C. These digests were cleaned using a C18 silica cartridge to remove the salt and then dried with a speed vac. The dried pellet was then resuspended in buffer A (5% acetonitrile, 0.1% formic acid). All the experiments were conducted using an EASY-nLC 1,000 system (Thermo Fisher Scientific) conjuction with Thermo Fisher-QEXACTIVE mass spectrometer equipped with nano-electrospray ion source. A 25 cm PicoFrit column (360 μm outer diameter, 75 μm inner diameter, 10 μm tip) packed with 1.8 μm of C18-resin (Dr Maeisch, Germany) was used to extract 1.0 μg of the peptide mixture. The peptides were loaded with buffer A and eluted at a flow rate of 300 nL/min for 100 min with a 0%–40% gradient of buffer B (95% acetonitrile, 0.1% formic acid). The top 10 data-dependent method was used for acquisition of MS data by considering the most abundant precursor ions from the survey scan.

### 2.8 Data processing

All samples were processed, and the RAW files generated were compared to the Uniprot HUMAN reference proteome (HUPO) database using Proteome Discoverer (v2.2). The precursor was set at 10 ppm and fragment mass tolerances was set at 0.5 Da for SEQUEST search. The protease used to generate peptides, with enzyme specificity set for trypsin/P (cleavage at the C terminus of “K/R: unless followed by “P”) and a maximum missed cleavages value of two. For database search, carbamidomethyl on cysteine was recognized as fixed modification, whereas N-terminal acetylation and methionine oxidation were classified as variable modifications. The peptides False Discovery Rate (FDR) and spectrum match was set to 0.01.

### 2.9 Quantitative proteomics

The following procedures were performed to execute Label free quantification (LFQ) suit for quantitative proteomics using MaxQuant v1.5.2.8 (http://www.maxquant.org/): feature identification, initial Andromeda search, recalibration, main Andromeda search, and posterior error probability calculation (likelihood of a protein being incorrectly recognized). At first, razor peptides and protein groups were identified. Proteins that cannot be identified unambiguously by distinct peptides but share peptides were grouped together and quantified as a single protein group. For instance, if all detected peptides of protein X were also identified for protein Y, X and Y are classified as one protein group (even though unique peptides were found for Y, since it was still uncertain whether X was present in the sample). Only one common quantification was generated for both proteins in the result. A Razor peptide is formed when two protein groups (protein A and protein B) are unequivocally identified by distinct peptides yet share a common peptide. Following the discovery of distinct and unique peptides, a “match across the runs” procedure was used to match the same accurate masses across several LC-MS/MS runs within a 1.5-min retention time frame. Relative quantification was determined by comparing the abundance of the same peptide species/protein across runs, whereas absolute quantification was determined by equating the quantities of various proteins in the same sample. The Summary statistics can be generated by the MaxQuant algorithms through peak detection and scoring of peptides, in addition to mass calibration and protein quantification. Protein abundances were normalised in MaxQuant using the LFQ technique and then transformed to Log2 for further analysis. The label-free method analyses the intensity of these peptides to determine peptide ratios by taking the greatest number of detected peptides between any two samples. The median values of all peptide ratios of a given protein are used to calculate protein abundance.

### 2.10 Bioinformatics analysis

The differentially expressed proteins (DEPs) calculated based on Log_2_ fold change > 1 and *p*-value < 0.05 were subjected to functional annotation and enrichment analysis by means of publicly available bioinformatics annotation tools and databases such as String, UniProt, and Cytoscape. Enriched terms were ranked by *p*-value (hypergeometric test) using Cytoscape ClueGO plugin. Venn diagram showing distribution pattern of proteins were drawn using Venny 2.1. The hierarchical clustering of DEPs between fertile donors and idiopathic infertile patients was analysed by creating heat map using R.3.4.4 package (Complex Heatmap map library). The Euclidean distance correlation matrix was employed for hierarchical clustering of the DEPs for dendrogram visualisation to illustrate comprehensive interaction between the proteins. The Biological Networks Gene Ontology (BiNGO) application in Cytoscape was used for the determination of significantly overrepresented Gene Ontology (GO) terms in the DEPs data set and the predominant functional themes of the tested DEPs were mapped to visualize the biological pathways altered in the infertile group. The STRING database (http://string-db.org/) was used to generate the protein-protein networks. A *p* < 0.05 was considered significant. Ingenuity pathway analysis (IPA), a proprietary curated database, was used to analyse the role of DEPs in biological and cellular processes, pathways, cellular distribution, protein-protein interactions, and regulatory networks.

### 2.11 Western blotting

Two key protein markers of PAH metabolism, i.e., AhR and HSP90β were validated by western blotting. Besides, the impact of induced oxidative stress on protein modifications, namely, 4-Hydroxynonenal (HNE) protein adduct formation and protein-tyrosine nitration were also studied by western blotting. The following primary antibodies were used for immunodetection, viz, anti-human AhR (sc-101104, Santacruz, Mouse), anti-human HSP90β (sc-59578, Santacruz, Mouse), anti-4-HNE (ab46545, Rabbit, Abcam) and anti-3-nitro-tyrosine (ab110282, Mouse, Abcam) For maintaining biological and technical variability, triplicate tests were performed on two individual and one pooled samples from each group. The protein concentrations of sample was normalized in each group. Spermatozoa were lysed in RIPA lysis solution (Sigma-Aldrich, St. Louis, MO, United States) with a proteinase inhibitor cocktail (Roche, Indianapolis, IN, United States) and kept overnight at 4°C.Samples containing 20–30 μg of protein in each well were separated on a 4%–20% SDS-PAGE and electroblotted onto polyvinylidenedifluoride (PVDF) membranes. The PVDF membrane was then blocked for 2 h with 5% non-fat dried milk in a Tris-buffered saline Tween 20 (TBST) buffer. Following that, the blots were incubated with primary antibodies overnight at 4°C, followed by the specified secondary antibodies (Mouse, Rabbit, Abcam) for 3 h at room temperature. The blots were then washed with TBST, and protein bands were detected using an enhanced chemiluminescence kit-Pierce™ ECL Western Blotting Substrate (Thermo Scientific, Rockford, IL, United States) in ChemiDoc™ MP Imaging System (BioRad, Hercules, CA, United States). Densitometric analysis of western blot pictures was done using the total intensity normalization method in Image Lab 6.0.1 (BioRad, Hercules, CA, United States). The results were expressed as a fold change in comparison to the fertile donor.

### 2.12 Statistical analysis

Statistical analysis was performed by MedCalc Statistical Software, ver. 17.4 (MedCalc Software; Ostend, Belgium). The data are expressed as mean ± standard deviation (SD). Normalization of data was assessed using Shapiro–Wilk test followed by Levene’s test for homogeneity of variance. The Mann-Whitney U-test was used to assess the data on sperm parameters and biochemical estimates. Welch’s *t*-test or unequal variances *t*-test was used to analyze the results of LC-MS/MS proteomics and Western blotting. A *p* < 0.05 (minimum) difference was considered as significant.

## 3 Results

### 3.1 Semen analysis

All idiopathic infertile patients included in this study have at least one semen parameter in semen analysis below (WHO, 2010) criteria. Although the average values were within the range, sperm concentration, motility, morphology and vitality were significantly different from the fertile donors. In the patient group, ∼30% have declined motility while ∼60% have above normal anomalous spermatozoa (Table 1).

**TABLE 1:** Semen parameters in fertile donor and Idiopathic infertile patients.

Parameter	WHO	Fertile donor	Idiopathic infertile patient	*p*-value
Volume (mL)	> 1.5	3.24 ± 0.49	3.03 ± 0.64	*p* = 0.0750
pH	7.2–8.0	7.39 ± 0.18	7.35 ± 0.17	*p* = 0.2806
Age	< 35	30.85 ± 3.02	30.71 ± 3.77	*p* = 0.1201
Sperm Motility (%)	> 40	56.30 ± 8.40	43.42 ± 11.37	*p* < 0.0001
Sperm Concentration (10^6^/mL)	> 15	123.33 ± 22.30	86.77 ± 40.88	*p* < 0.0001
Sperm viability (%)	> 58	53.45 ± 10.23	39.75 ± 9.45	*p* < 0.0001
Sperm morphology (%)	> 4	12.5 ± 1.5	20.5 ± 5.3	*p* < 0.0001

### 3.2 PAH exposome in semen

**FIGURE 1:**
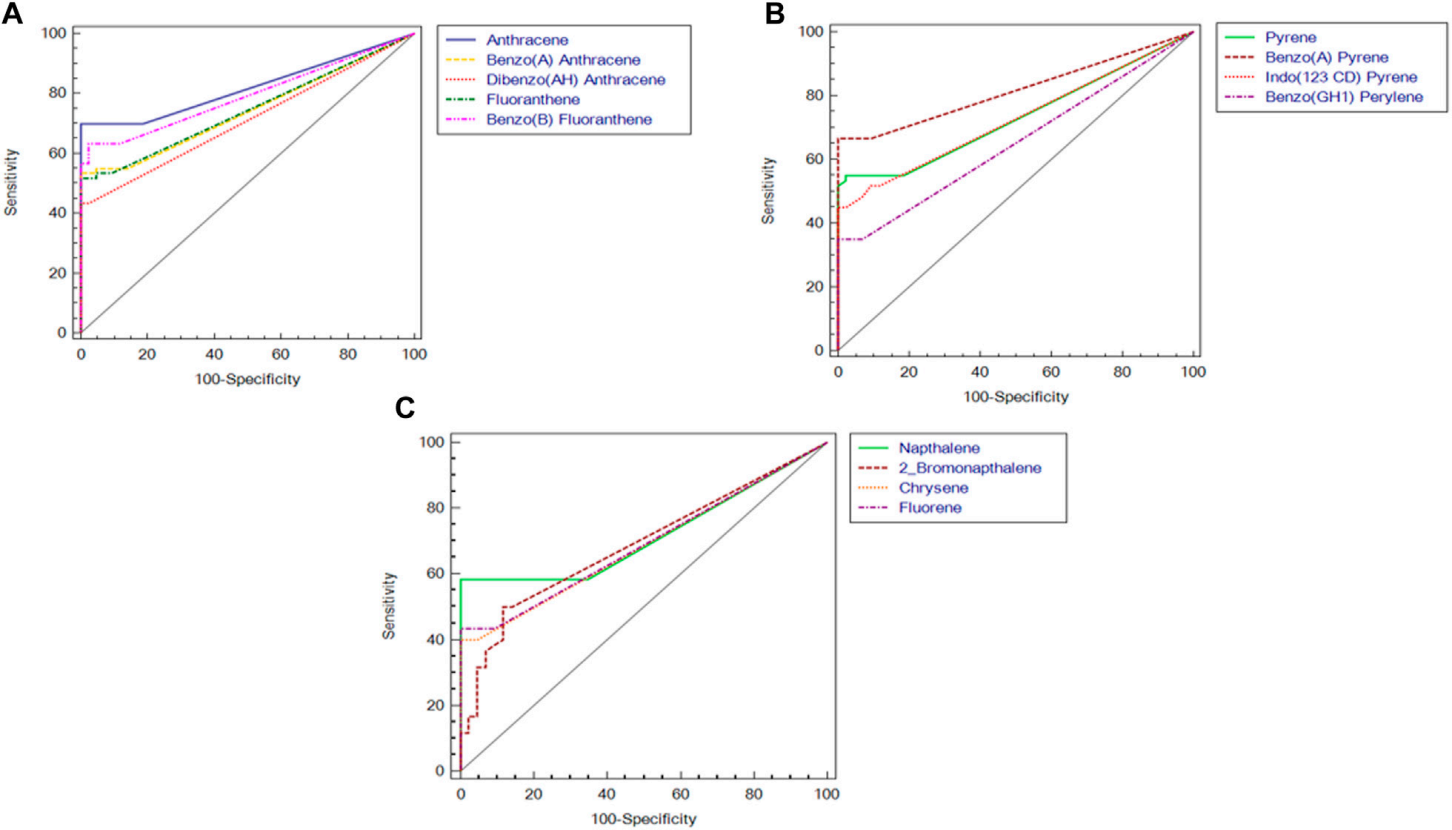
Receiver Operating Characteristic (ROC) curves of polyaromatic hydrocarbons in semen of idiopathic infertile men (*n* = 60) in comparison to fertile donor (*n* = 43). **(A)** Anthracene, Benzo(a)anthracene, dibenzo[a,h]anthracene, fluorathene, benzo[b]fluorathene; **(B)** pyrene, benzo(a)pyrene, indo[1,2,3-c,d]pyrene, benzo[g,h,i]perylene; **(C)** naphthalene, 2-bromo naphthalene, chrysene, fluorene.

Out of the 16 standard PAHs used for screening, a total of 13 PAH metabolites, i.e., Anthracene, Benzo (A) Anthracene, Benzo (A) Pyrene, Benzo (B) Fluoranthene, Benzo (GH1) Perylene, Chrysene, Dibenzo (AH) Anthracene, Fluorene, Fluoranthene, Indo (123 CD) Pyrene, Napthalene, 2 Bromonapthalene, Phenanthrene, Pyrene were detected in the semen samples at ng/mL level (Table 2). However, the concentration of PAH in the semen of idiopathic infertile patients were significantly higher in comparison to the fertile donor. The cut-off level of these PAHs (if any) determined by ROC curve analysis segregates the fertile from the idiopathic infertile patients. The results of the ROC analysis are presented in Figure 1; Supplementary Table S1. Benzo (a) Pyrene particularly is found to be highly discriminative among 13 PAHs in idiopathic infertile patients.

**TABLE 2:** Concentration of PAH in the semen of fertile donor and idiopathic infertile patients. Data are expressed as mean ± SD.

Sl. No.	PAH (ng/mL)	Fertile donor (*n* = 43)	Idiopathic infertile patient (*n* = 60)	Mann whitney *U* test (*p*-value)
1	Anthracene	9.00 ± 19.59	415.60 ± 327.97	*p* < 0.0001
2	Benzo (A) Anthracene	20.79 ± 66.24	509.97 ± 486.47	*p* < 0.0001
3	Benzo (A) Pyrene	0.35 ± 1.17	43.37 ± 38.57	*p* < 0.0001
4	Benzo (B) Fluoranthene	3.60 ± 12.58	83.52 ± 105.75	*p* < 0.0001
5	Benzo (GHI) Perylene	7.26 ± 26.82	196.10 ± 291.58	*p* = 0.0005
6	Chrysene	0.26 ± 1.18	15.77 ± 22.30	*p* < 0.0001
7	Dibenzo (AH) Anthracene	0.16 ± 1.07	16.15 ± 20.81	*p* < 0.0001
8	Fluorene	49.67 ± 169.37	1857.60 ± 2,381.74	*p* = 0.0001
9	Fluoranthene	1.09 ± 3.78	27.58 ± 29.77	*p* < 0.0001
10	Indol (123CD) Pyrene	0.74 ± 2.13	7.32 ± 8.00	*p* < 0.0001
11	Napthalene	199.65 ± 299.65	1752.88 ± 1728.33	*p* = 0.0001
12	2Bromonapthalene	68.21 ± 185.99	317.62 ± 385.50	*p* = 0.0001
13	Pyrene	4.40 ± 11.68	54.33 ± 54.68	*p* < 0.0001

### 3.3 Effect of PAH concentration on sperm redox status

**FIGURE 2:**
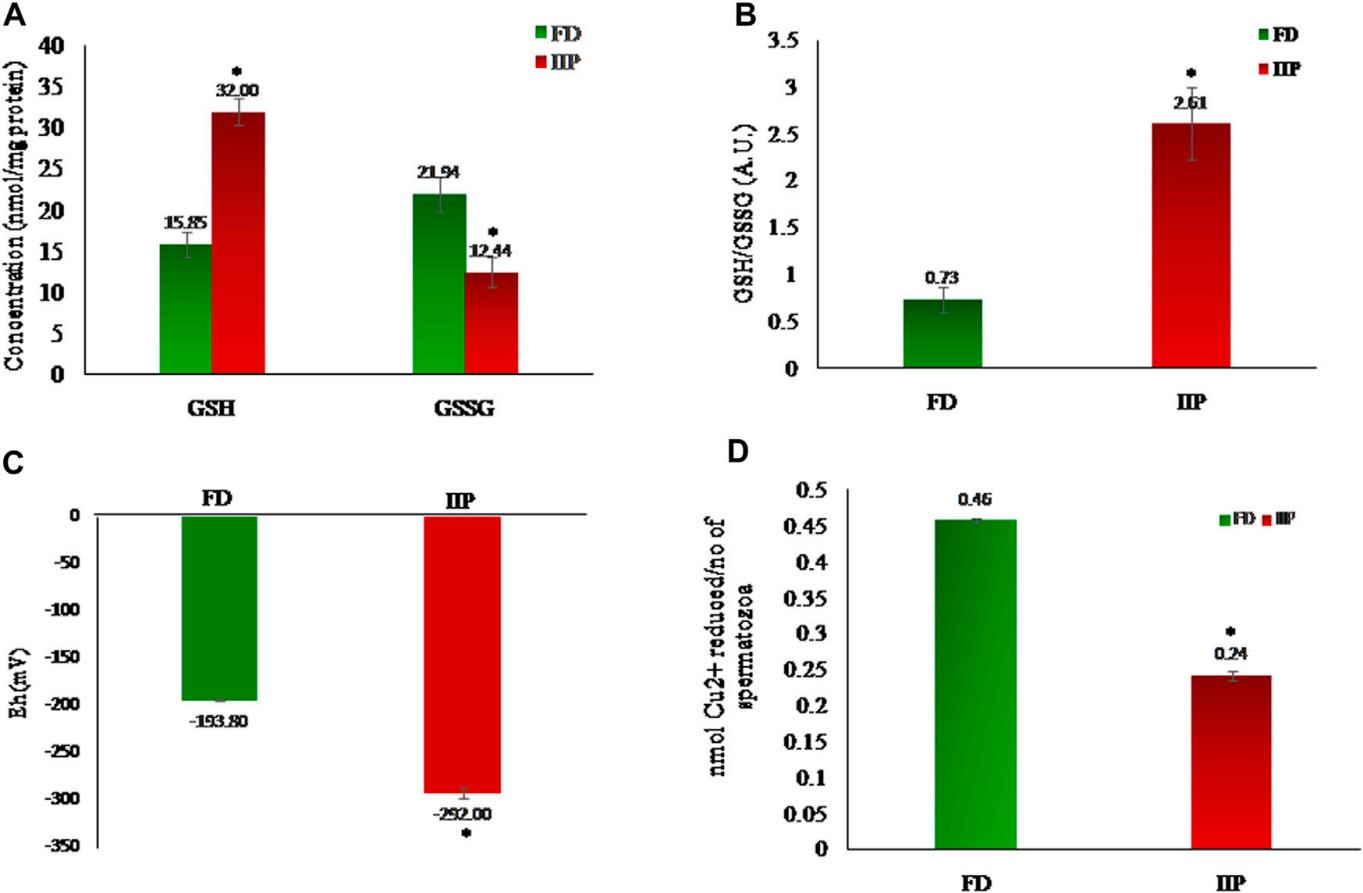
Comparison of redox status in the spermatozoa of fertile donor (*n* = 43) and infertile patients (*n* = 42). **(A)** Levels of reduced (GSH) and Oxidized glutathione (GSSG); **(B)** Spermatozoal redox status; **(C)** Half-cell reduction potential. **(D)** Total antioxidant capacity. FD: Fertile donors; IIP: Idiopathic infertile patients. Data are expressed as mean ± SD. *p < 0.05.

To corroborate the alteration in the redox environment of spermatozoa in the idiopathic infertile patients, the ratio of GSH:GSSG was measured. An increase in the absolute concentrations of GSH and GSH:GSSG ratio was noticed in idiopathic infertile patients (Figures 2A, B). The reduction potential in spermatozoa of idiopathic infertile patients is more negative with respect to fertile donor (Figure 2C). A reduction in the level of total antioxidant capacity of sperm was observed in the infertile group as compared to fertile control (Figure 2D).

### 3.4 Global proteome profiling of spermatozoa

**FIGURE 3:**
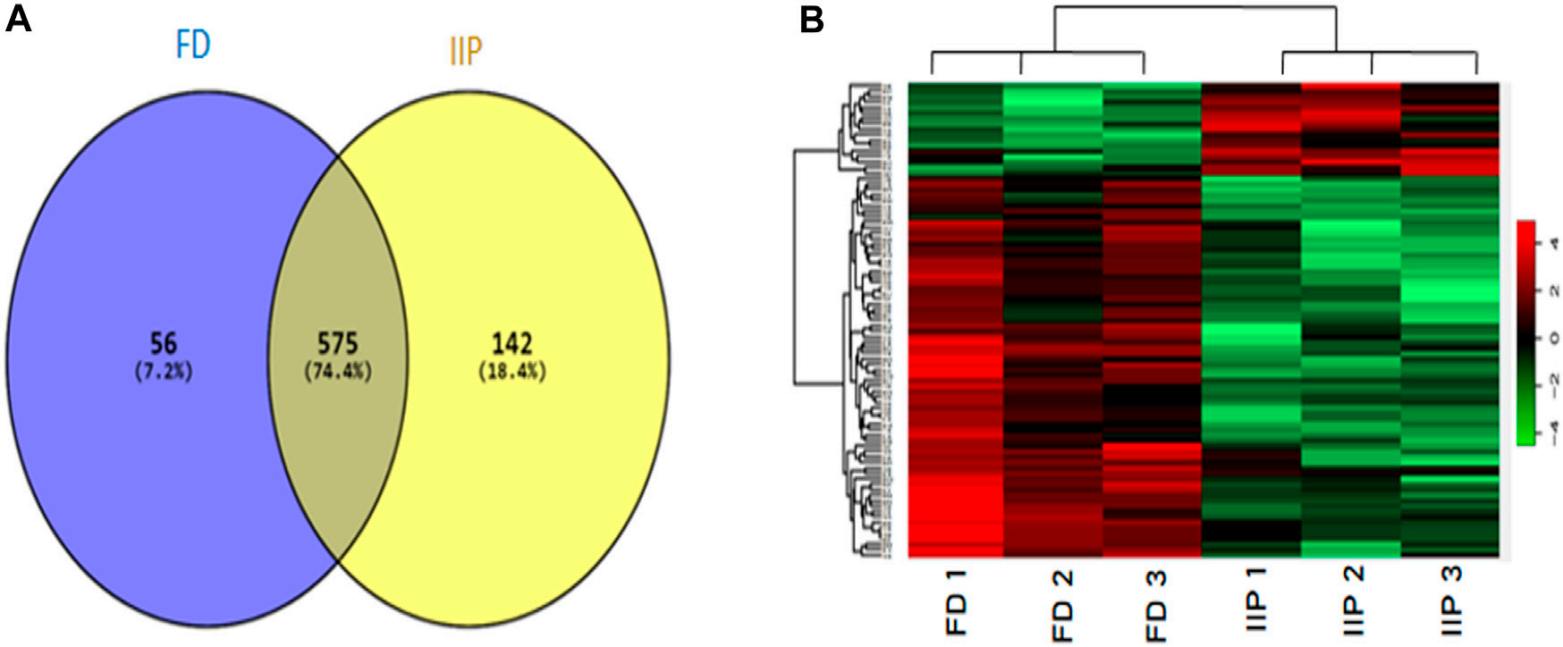
Comparative global proteomic profiling of spermatozoa between FD: Fertile Donors and IIP: Idiopathic infertile patients. **(A)** Venn diagram showing distribution of differentially expressed proteins (DEPs) **(B)** Heat map showing a hierarchical cluster of DEPs. The dendrogram for sample replicates (column clustering) separated the samples according to their clinical diagnosis into FD and IIP. Hierarchical clustering analysis between protein expression profiles of DEPs (row clustering) separated overexpressed DEPs in IIP from underexpressed DEPs in FD. The green and red color denoted low and high expression levels respectively as shown in attached graduated color scale bar. Log_2_ fold change > 1 and *p*-value < 0.05.

The quantitative differential proteomic analysis identified a total of 773 proteins in fertile donor and idiopathic infertile patients by label free LC-MS/MS. Out of the total 773 proteins, 631 were from fertile donor and 717 from idiopathic infertile patients with 575 proteins common in both (Figure 3). A total of 71 DEPs (based on Log_2_ fold change > 1 and *p*-value < 0.05) were detected, of which 13 and 58 were under- and over-expressed, respectively in idiopathic infertile patients compared to fertile donor (Figure 3; Supplementary Table S2). The hierarchical clustering by heat map showed over and under expressed proteins into the two groups distinctively (Figure 3).

**FIGURE 4:**
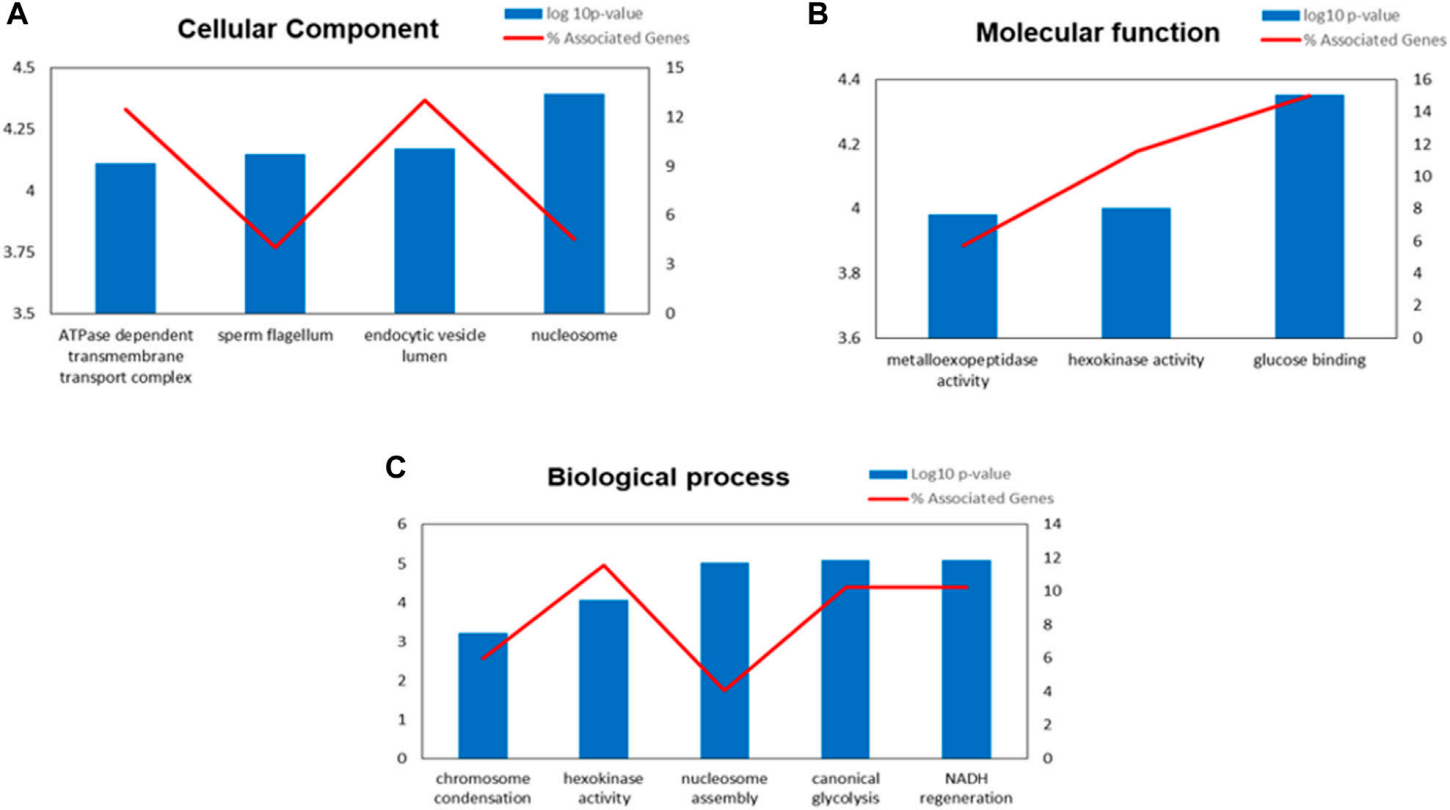
Gene Ontology (GO) enrichment analysis result of differentially expressed proteins (DEPs) in IIP: Idiopathic infertile patients compared to FD: fertile donor. Bar graph showing the top GO terms for cellular component **(A)** molecular function **(B)** and biological process **(C)**.*p < 0.05.

The functional enrichment analysis of Gene Ontology (GO) by ClueGO revealed that the identified proteins were involved in various crucial biological functions such as chromosome condensation (GO:0030261), hexokinase activity (GO:0004396), nucleosome assembly (GO:0006334), canonical glycolysis (GO:0061621) and NADH regeneration (GO:0006735). The enriched cellular component and molecular functions were ATPase dependent transmembrane transport complex (GO:0098533), sperm flagellum (GO:0036126), endocytic vesicle lumen (GO:0071682), nucleosome (GO:0000786), metallo-exopeptidase activity GO:0008235, hexokinase activity (GO:0004396) and glucose binding (GO:0005536). The enriched processes and the identified proteins involved in various molecular functions along with its localisation were shown in Figure 4; Supplementary Table S3.

### 3.5 Functional pathway analysis of differentially expressed protein

**FIGURE 5:**
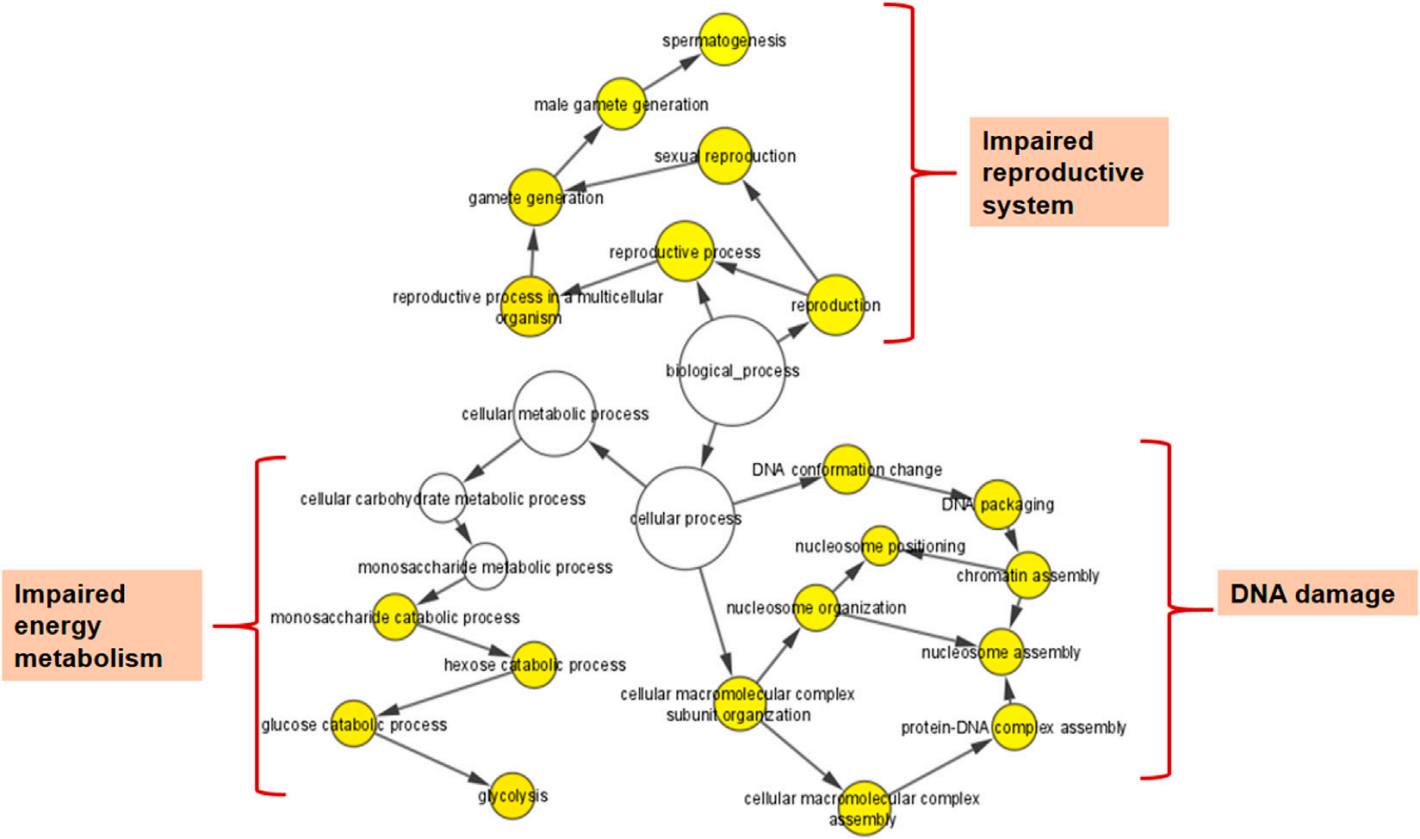
Cytoscape (BiNGO app) enrichment analysis revealed over-represented biological processes for the differentially expressed proteins (DEPs) in the spermatozoa of idiopathic infertile patients in comparison to fertile donor.*p < 0.05.

**FIGURE 6:**
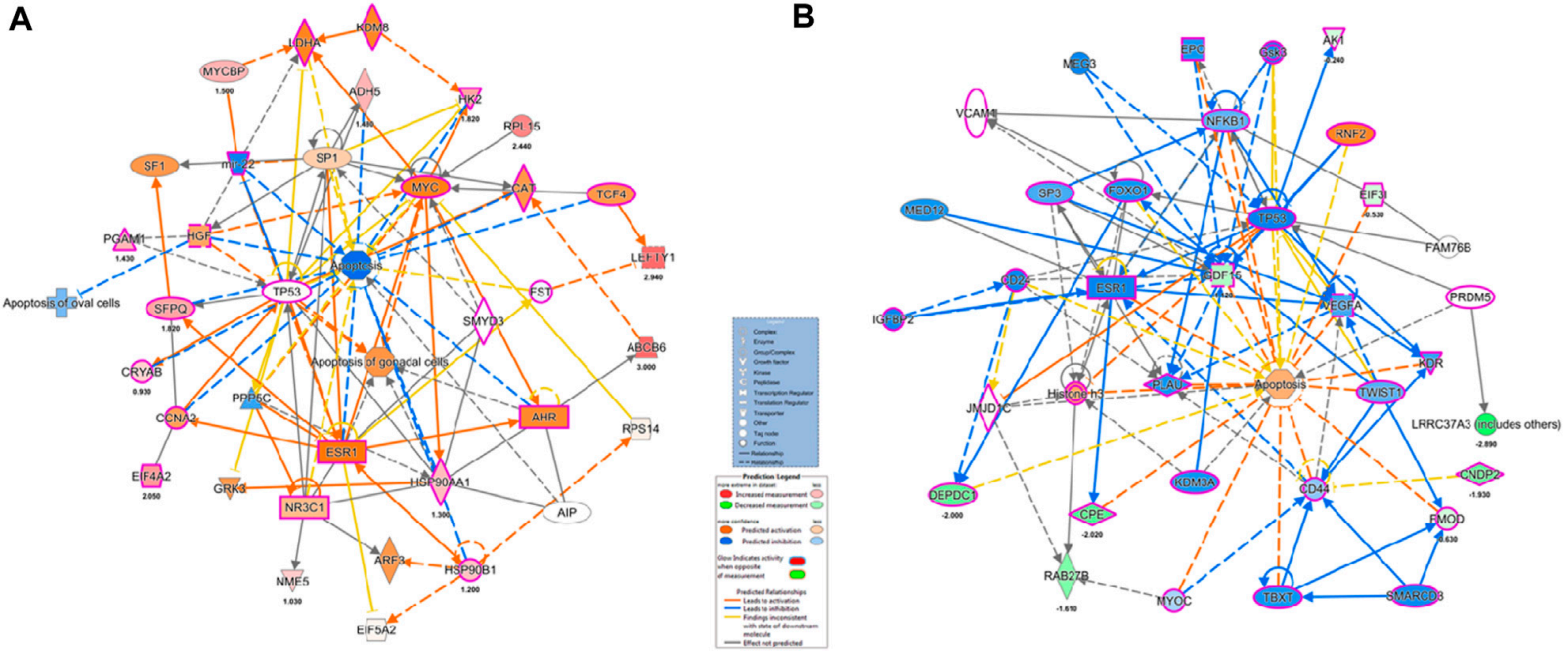
Ingenuity pathway Analysis of overexpressed and under-expressed proteins in Idiopathic infertile patients compared to fertile donor top Disease and Function **(A)** Cancer, Endocrine System Disorders, Organismal Injury and Abnormalities **(B)** Cancer, Cell Death and Survival, Organismal Injury and abnormalities, respectively. *p < 0.05.

The BiNGO mapping revealed the involvement of DEPs in reproduction, spermatogenesis, nucleosome assembly, chromatin assembly, DNA packaging and glycolysis (Bonferroni step down with *p* value ≤ 0.001) resulting in DNA damage, impaired energy metabolism and reproductive function (Figure 5). String protein-protein interaction analysis of DEPs revealed that the major pathways deregulated are Glycolysis/Gluconeogenesis (HAS:00010; FDR 1.15e-06), Fatty acid degradation (HAS:00071; FDR 0.0054),HIF-1 signaling pathway (HAS:04066; FDR 0.0281), Estrogen signaling pathway (HAS:04915; FDR 0.0498), Oxidative phosphorylation (HAS:00190; FDR 0.0498), Metabolic pathways (HAS:01100; FDR 0.0029), DNA packaging (GO:0006323; FDR 0.00072), Regulation of regulation of reactive oxygen species metabolic process (GO:2000377; FDR 0.0414), Post-translational protein modification (GO:0043687; FDR 0.0080), and Spermatogenesis (GO:0007283; FDR 0.0284) (Supplementary Figure S1). The protein interaction of upregulated DEPs by IPA identified the topmost molecular network to be associated with Cancer, Endocrine System Disorders, Organismal Injury and Abnormalities where out of the 35 nodal proteins 18 were detected in our dataset. In the second most pathway the proteins were involved in Cell Death and Survival, Cellular Development, Organismal Survival where out of 21 nodal proteins 12 were from our dataset. The downregulated DEPs topmost network is associated with Cancer, Cell Death and Survival, Organismal Injury and Abnormalities where out of 19 nodal proteins 9 were found in our dataset (Figure 6; Supplementary Table S4).

**FIGURE 7:**
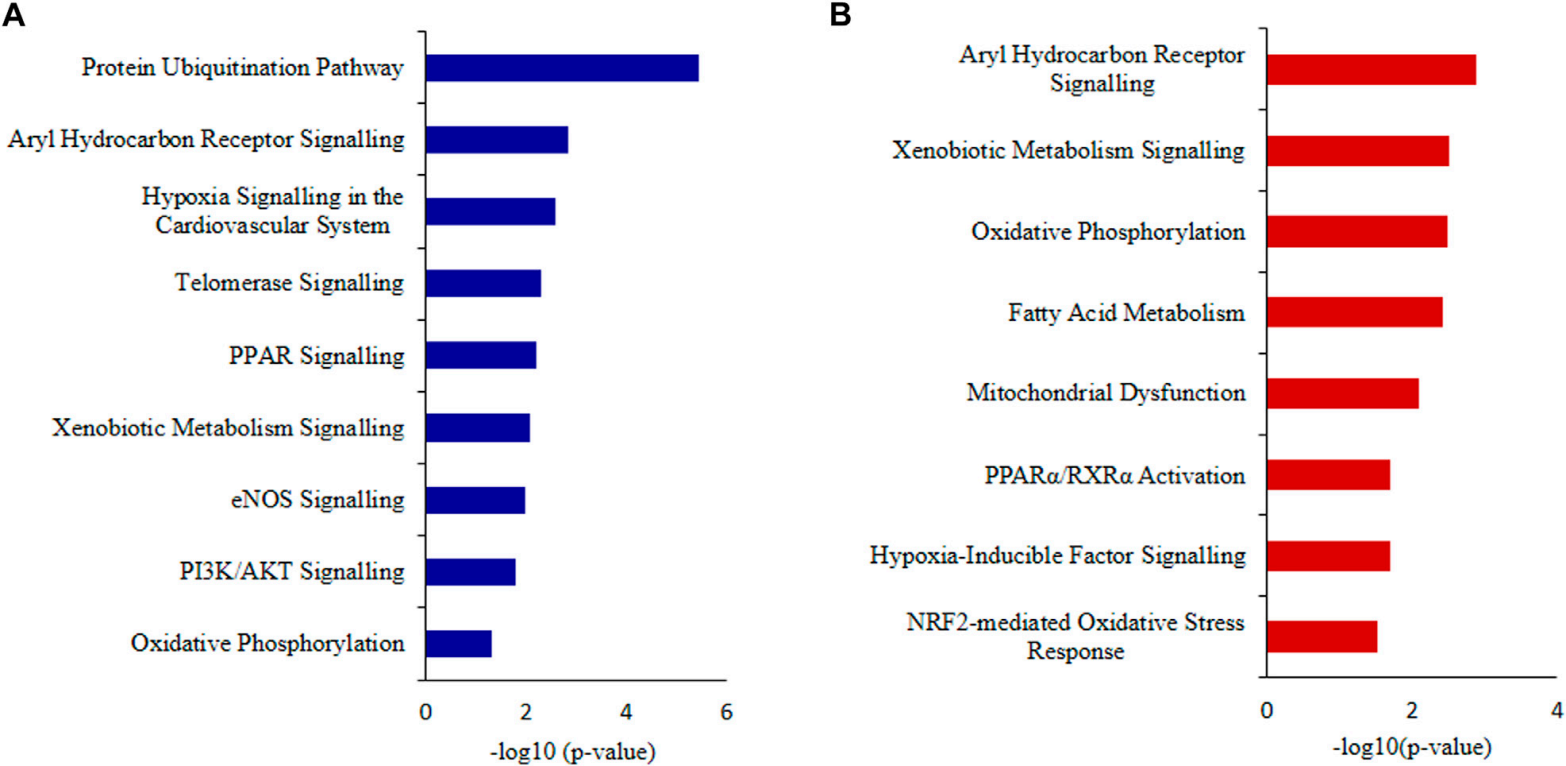
Ingenuity Pathway Analysis (IPA) **(A)** Canonical pathways analysis and **(B)** toxicity lists analysis of the differentially expressed proteins (DEPs) of idiopathic infertile patients in comparison to fertile donors. *p < 0.05.

IPA canonical pathway revealed that Aryl Hydrocarbon Receptor Signaling, Hypoxia Signaling in the Cardiovascular System, Telomerase Signaling, PPAR Signaling, Xenobiotic Metabolism Signaling, eNOS Signaling and Oxidative Phosphorylation were deregulated (Figure 7A; Supplementary Table S5).

The top toxicity list and functions determined by IPA-Toxicological pathway showed that Aryl Hydrocarbon Receptor (AhR) Signalling, Xenobiotic Metabolism Signalling, Fatty Acid Metabolism, Hypoxia-Inducible Factor Signalling, NRF2-mediated Oxidative Stress Response, Mitochondrial Dysfunctionand Oxidative Phosphorylation were the most affected toxicological functions (Figure 7B; Supplementary Table S6).

### 3.6 Expression profile of key pathway proteins

**FIGURE 8:**
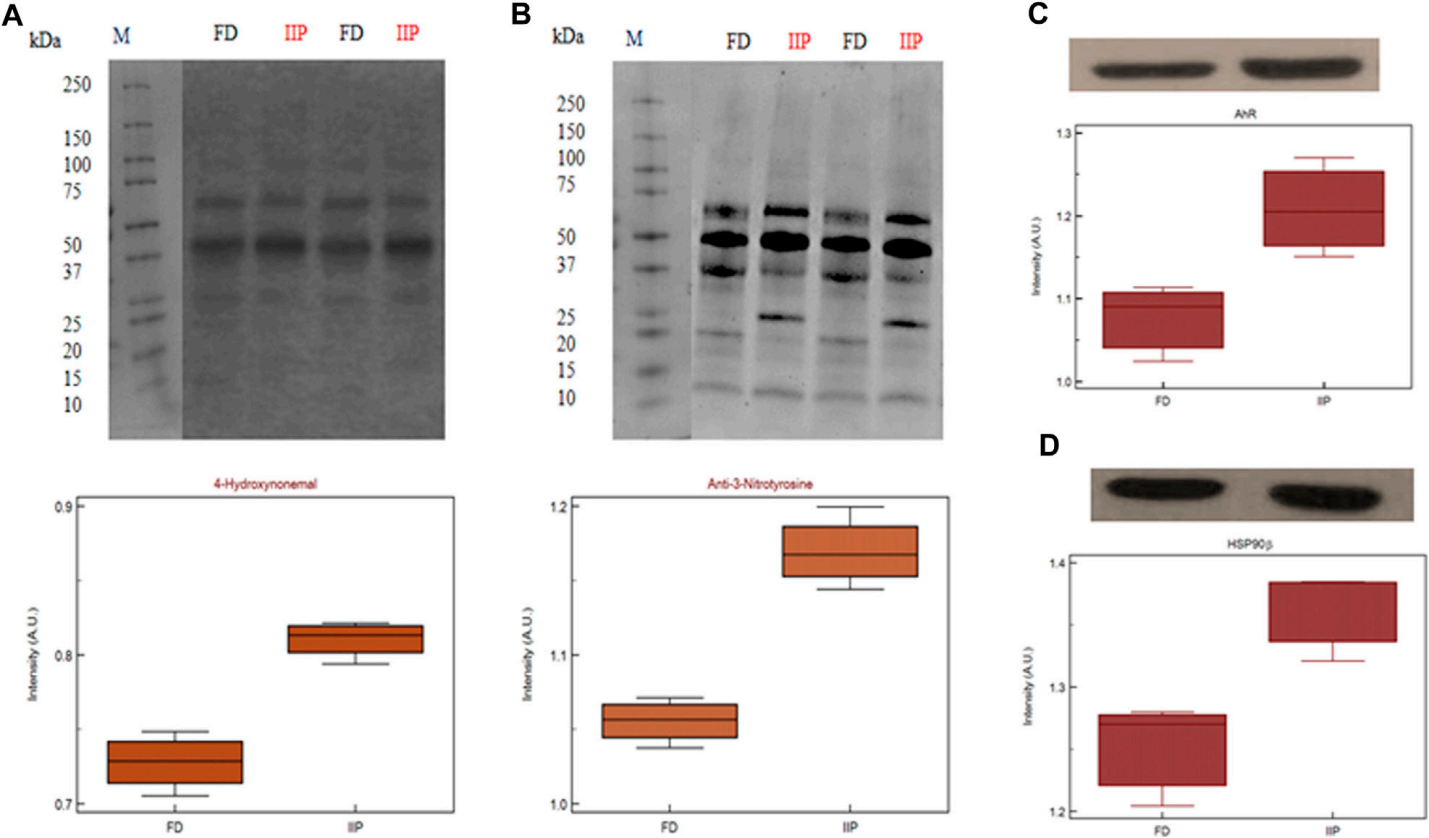
Expression profile of **(A)** 4-Hydroxynonenal **(B)** 3-Nitrotyrosine **(C)** AhR **(D)** HSP90β and their respective densitometry analysis in spermatozoa of FD: fertile donor and IIP: idiopathic infertile patient with total protein normalization (in arbitrary unit). *p < 0.05 with respect to fertile donor.

The key pr**o**teins predicted in top canonical pathway, AhR and Heat shock protein (HSP)90β validated by western blot (Figures 8C, D) which corroborated the LC-MS/MS data. Both the proteins were found to be over expressed in the idiopathic infertile patients as compared to fertile donors. The expression of 4-Hydoxynonenal (HNE) protein adducts and protein nitrosylation were also found to be augmented in idiopathic infertile patients (Figures 8A, B).

## 4 Discussion

Polyaromatic hydrocarbons (PAHs) are known endocrine disruptors which mimic the reproductive hormones and interfere with their synthesis by acting as agonist and antagonists. Several research studies on adult rodents with PAHs such as B(a)P, 2,3,7,8-Tetrachlorodibenzodioxin and 3-Methylchloranthrene resulted in an increase in the number of abnormal sperm and immature germ cells (Viczian, 1968; Wyrobek and Bruce, 1975), affected spermatogenesis by causing testicular atrophy (Mattison, 1982), diminished testicular weight and increased apoptosis in seminiferous tubules (Denison and Heath-Pagliuso, 1998; Coutts et al., 2007). Apart from these observations, PAHs also disrupt the normal embryonic development by inducing oxidative stress. A study by Delfino et al. (2011) demonstrated that exposure to PAHs upset the redox balance and generate reactive oxygen species (ROS). This leads to oxidative stress causing damage to biomolecules such as DNA, lipid and protein involved in the development of reproductive process. In the current study, PAH concentration was measured in the semen of idiopathic infertile patients followed by proteomics of spermatozoa to understand the mechanism(s) by which PAHs elicit male infertility. Several studies have shown that urinary 1-hydroxypyrene (1-OHP) is a good biological index for the occupational exposure assessment of PAHs (Xia et al., 2009). In a study by Xia et al. (2009) men with higher urinary concentrations of 1-hydroxypyrene, 2-hydroxyfluorene and sum of all four PAH metabolites (assessed as tertiles) were more likely to have idiopathic male infertility. In second study by the same authors (Xia et al., 2009) opined higher urinary 1-hydroxypyrene (assessed as quantiles) levels to be a potential cause for below reference sperm concentration and total sperm count. Similarly, Song et al. (2013) found a direct correlation between blood concentrations of PAH with sperm motility and a decrease in pregnancy outcome. But no concrete data were available on semen concentration of PAH with respect to infertility to justify their involvement in spermatogenesis, sperm maturation and sperm function.

This pioneer study reports association between idiopathic male factor infertility with comprehensive screening data of PAHs in the semen resulting in sperm dysfunction through shotgun proteomic analysis. A total of 13 PAHs out of the 16 standards were identified in our sample implying the ubiquitous incidence of PAH in the semen irrespective of occupational exposure. The concentrations of all the 13 PAHs detected were significantly higher (*p* < 0.0001) in idiopathic infertile patients with respect to fertile donors. Based on AUC_ROC_ the PAHs having most significant effect on fertility are of the following order Anthracene < benzo(a)pyrene < benzo [b]fluoranthene < Fluoranthene < benzo(a)anthracene < indol (123CD)pyrene < pyrene < naphthalene < dibenzo (AH)anthracene < fluorine < 2bromonaphthalene < chrysene < benzo (GH1)perylene (Figure 1; Supplementary Table S1). Irrespective of fertility status all analyzed samples possessed naphthalene, albeit at different concentration showing the highest cut-off value of 868 ng/mL. On the other hand, the lowest was noticed for both chrysene and benzo(a)pyrene at 6 ng/mL and benzo(a)pyrene being the ubiquitous one in idiopathic infertile patients distinctively segregating infertile men from their fertile counter parts. Four out of 43 semen samples analyzed in fertile donor (∼9%) showed measurable benzo(a)pyrene (0.35 ± 1.17 ng/mL). On the other hand, substantially high level of benzo(a)pyrene (43.37 ± 38.57 ng/mL) was detected in all the 60 semen samples analyzed in idiopathic infertile patients. Thus, benzo(a)pyrene can be used as a marker to distinguish infertile men from fertile one with 66.67% sensitivity and 100% specificity at 95% CI (confidence interval). A large cohort study may further substantiate our findings. Though most of the idiopathic infertile males participated in the present study have at least one sperm parameter abnormal, the cumulative spermiogram shows values with WHO, 2010 range. However, ∼30% samples showed declined motility while∼60% have above normal anomalous spermatozoa. It will not be out of context to mention that prenatal exposure of benzo(a)pyrene to Gclm knockout mice resulted reduction in testicular weight, testicular sperm head counts, epididymal sperm counts, and epididymal sperm motility when analyzed at 10-weeks of age, relative to wild type littermates (Nakamura et al., 2012). In another study on Mexican workers in a rubber factory with potential occupational exposure to PAHs, impaired spermatogenesis was reported evinced by increased anomalies in sperm concentration, motility and morphology (De Celis et al., 2000). In fact, we have observed increased retention of histone proteins (H1-3, H1-4, H1-7, H2BC19P, H2BC11, H2BC12, H2BS1) in the spermatozoa of idiopathic infertile patients implying improper nuclear remodelling (Torres-Flores and Hernandez-Hernandez, 2020). Furthermore, the gene ontology and protein-protein interaction analysis data (BiNGO, ClueGO, String) reveal that DNA packaging, chromatin assembly and nucleosome assembly is deregulated in patient group. PAHs are also known to cause potential DNA damage in case of idiopathic infertile males (Saad et al., 2019). Interactive metabolites of PAHs may reach the testicles and epididymis forming sperm DNA adducts (Gaspari et al., 2003). In addition, the compounds resulting from PAH oxidation have the ability to enter oxidation cycles, which increase the generation of ROS and thus cause sperm DNA damage. To corroborate the findings we observed significantly higher 4-HNE and S-Nitrotyrosine levels in the spermatozoa of infertile patients in comparison to their fertile counterparts. Therefore, it may be inferred that, apart from improper compaction and packaging of sperm DNA, the major alterations in carbohydrate metabolism and active transport across membrane lead to production of dysfunctional spermatozoa. The predicted altered NADH regeneration pathway and the monitored glutathione redox status further corroborates the imbalance in cellular redox state which is expected from redox acting toxicants like PAHs (Urlacher and Eiben, 2006).

In this study, 71 DEPs were reported in patient group with higher levels of PAH and IPA toxicity list of these DEPs was predicted to be involved in Aryl Hydrocarbon Receptor (AhR) Signalling, Xenobiotic Metabolism Signalling, Hypoxia-Inducible Factor Signalling, NRF2-mediated Oxidative Stress Response, Mitochondrial Dysfunction and Oxidative Phosphorylation. The AhR is ligand-activated transcription factor that responds to endogenous ligands in addition to exogenous xenobiotic ligands, such as PAHs (Denison et al., 2011). Upon ligand binding, AhR translocates to nucleus where it binds to AhR nuclear transporter (ARNT) and activates xenobiotic metabolizing enzymes: cytochrome P450 (CYP) 1A1, 1A2, and 1B1 for catalyzing oxidative biotransformation of xenobiotics (Khorram et al., 2004; Zanger and Schwab, 2013). After biotransformation PAHs generate potential reactive intermediates (Shimada and Fujii-Kuriyama, 2004; Shimada et al., 2013). In fact, Hansen et al. (2014) reported the role of AhR signalling in maintenance of Sertoli cell architecture and resultant spermatogenesis in AhR knockout mice where the poorly remodelled spermatozoa are suggested to be more susceptible to oxidant attack. In the present study an increased expression of 4-HNE and 3-Nitrotyrosine implies induction of oxidative stress. On the other hand, 4-HNE is known to produce DNA adduct (Bin et al., 2016) as observed in case of PAH exposure and AhR signalling (Gu et al., 2012). It is pertinent to mention here that the levels of 4-HNE within spermatozoa are positively correlated with mitochondrial superoxide formation (Aitken et al., 1989), and elevated 4-HNE is responsible for numerous adverse effects on sperm function such as decline in motility, morphology, acrosome reaction, sperm-oocyte interaction and apoptosis (Aitken et al., 2012a; Aitken et al., 2012b). The BiNGO and IPA canonical pathway analysis further supports the hypothesis. Protein S-nitrotyrosination is responsible for protection of the proteins under oxidative stress, however irreversible S-nitrotyrosination leads to pathological condition. Of late, it has been elucidated that hydrophobic bio-structures like cell membranes and lipoproteins undergo S-nitrosylation and has strong association with lipid peroxidation (Bartesaghi and Radi, 2018). Therefore, it is quite natural to observe an increase in both 4-HNE and 3-nitrotyrosine concentrations in the spermatozoa of infertile patients implying PAH-induced oxidative stress. Further, experimental strategies may reveal the proximal oxidizing mechanism during tyrosine nitration including mapping and identification of the tyrosine nitration sites in specific proteins in the spermatozoa of idiopathic infertile men. Moreover, parallel over-expression of AhR and HSP90β as observed by western blot in the present study corroborated the finding as it is suggested that ligand-bound AhR translocates to the nucleus with HSP90β showing its co-localization in the nucleus (Tsuji et al., 2014). In contrast to AhR-dependent and CYP1A-mediated production of intracellular ROS, the AhR signaling pathway also regulates the expression of genes involved in antioxidant responses. Apart from AhR signalling, NRF2 is a key transcription factor that regulate genes involved in the metabolism of xenobiotics and endogenous chemicalsis reported as the top toxicological pathway in our DEP data set. Both signaling pathways react to environmental and endogenous stimuli. Despite the fact that AhR and NRF2 are clearly distinct signaling pathways, recent reports demonstrate the cross-regulation between these two signaling axes implying an integrated response to environmental stressors (Kohle and Bock, 2006).

Glutathione, an important antioxidant is involved in the elimination of PAHs and spermatozoa depend heavilyon glutathione metabolizing system for its survival (Adeoye et al., 2018). The reactive intermediates formed after metabolism of PAHs are conjugated to glutathione and eliminated by glutathione-S-transferases (GST) and glutathione peroxidase (GPx) (Penning et al., 1996). Glutathione (GSH) acts as a redox sensor by oxidizing to glutathione disulfide molecule (GSSG). So the ratio of GSH to GSSG is used as a biosignature of oxidized intracellular environment (Lertratanangkoon et al., 1997). A recent report by Branco et al. (2021) have reported that PAH and their metabolites show idiosyncratic behavior with respect to glutathione metabolism where phenanthrene induced higher ROS production. On the other hand, the authors reported increased GSH levels by benzo(b)fluoranthene along with augmented levels of protein sulfydryl group. The upregulation of GSH was opined to be a consequence of Nrf2 signalling activation and increased levels of glutathione metabolising enzymes and their mRNA after exposure to benzo(b)fluoranthene, but not during exposure to phenanthrene (Branco et al., 2021). As previously reported by our team in patients with unilateral varicocele (Swain et al., 2020), evidence from the current investigation demonstrates that spermatozoa from infertile men are in an energy-depleted and hypoxic condition as a result of their decreased redox potential.

## 5 Conclusion

**FIGURE 9:**
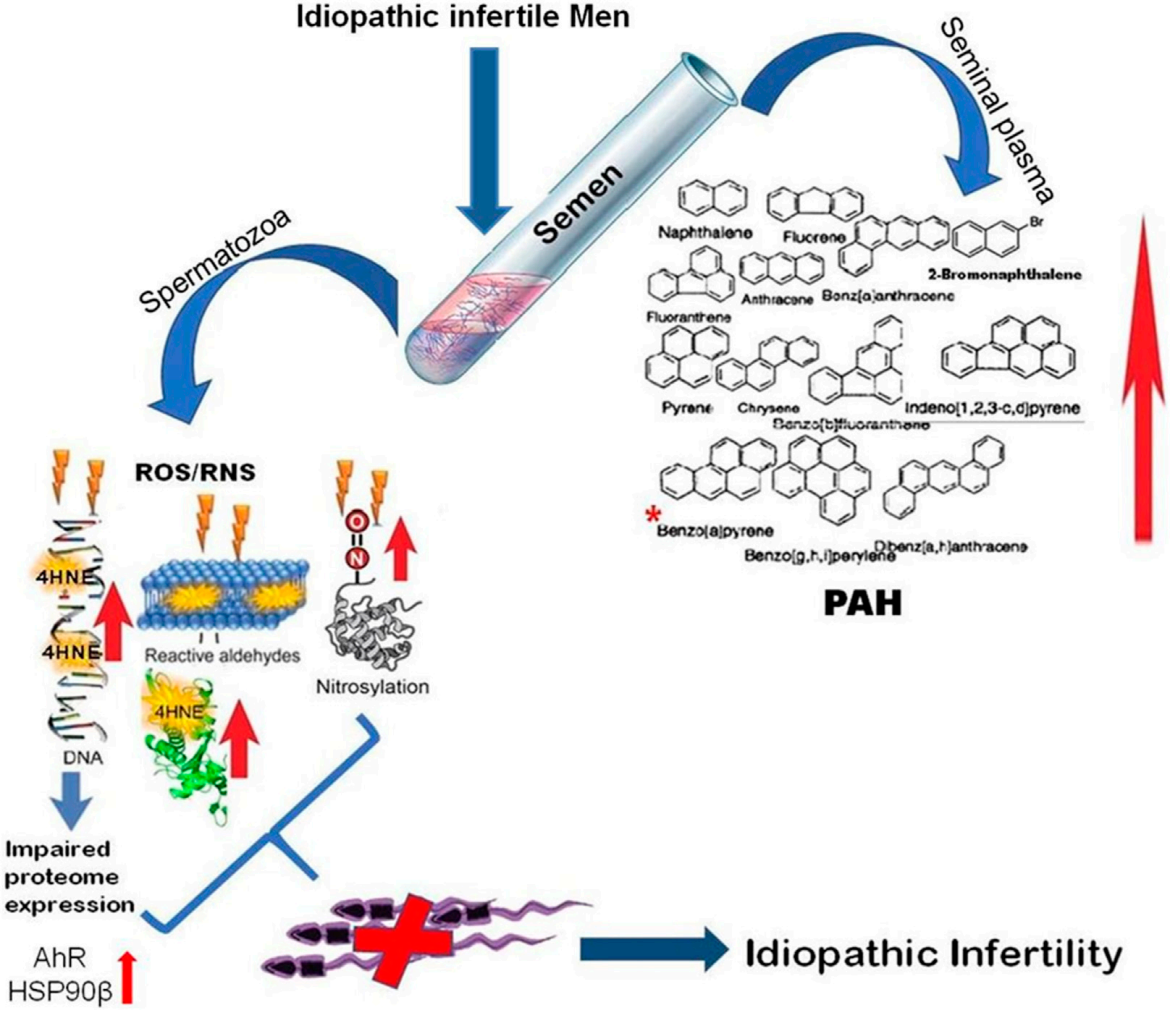
Schematic representation of molecular mechanisms involved in environmental borne polyaromatic hydrocarbon induced sperm dysfunction in idiopathic male infertility.

The present findings surmise the adverse impact of environment borne PAHs exposure on sperm function in idiopathic infertility which are largely ignored in regular infertility assessment. The high level of benzo(a)pyrene in the infertile group could serve as a predictive marker for idiopathic infertility along with the signature proteins AhR and the HSPs, particularly the HSP90. The presence of oxidative protein modification and differential expression of proteins involved in chromatin packaging and DNA damage further corroborates the noxious effect of PAH in semen (Figure 9). Therefore, it is suggested that along with seminogram and other biological markers, analysis of seminal levels of environmental toxins such as PAH in general and benzo(a)pyrene in particular may help in proper management of idiopathic male factor infertility.

## Data Availability

The data presented in the study are deposited in the ProteomeXchange repository, accession number PXT041615.
